# Clinical outcome of rim-plate-augmented separate vertical wiring with supplementary fixation for the treatment of patellar fracture associated comminuted inferior pole

**DOI:** 10.1038/s41598-023-40417-w

**Published:** 2023-08-18

**Authors:** Won-Tae Cho, Seungyeob Sakong, Jung Sunwoo, Wonseok Choi, Yun-Ki Ryu, Jeong-Seok Choi, Jong-Keon Oh, Beom-Soo Kim, Jae-Woo Cho

**Affiliations:** 1 Department of Orthopaedic Surgery, Ajou University Hospital, School of Medicine, Ajou University, Suwon, Republic of Korea; 2 Department of Orthopaedic Surgery, Korea University Guro Hospital, Korea University Medicine, 148, Gurodong-Ro, Guro-Gu, Seoul, 08308 Republic of Korea; 3 Department of Orthopaedic Surgery, Keimyung University Dongsan Hospital, School of Medicine, Keimyung University, 1035, Dalgubeol‑daero, Dalseo‑gu, Daegu, 42601 Republic of Korea

**Keywords:** Health care, Medical research

## Abstract

Despite the variety of treatment methods, comminuted inferior pole fractures of the patella remain difficult and technically demanding to achieve stable internal fixation. The purpose of this study is to evaluate the clinical outcomes of rim plate-augmented separate vertical wiring with supplementary fixation in the management of comminuted inferior pole fractures, AO/OTA 34-A1, C2, and C3, which has the secondary horizontal fracture line on lower articular boundary. From our study, bony union was achieved in all patients at an average of 3.1 ± 1.4 months after surgery. There was no patient with loss of reduction, fixation failure, or infection during follow-up. The average final range of motion was 131.6° ± 7.2°. Lysholm knee scores gradually increased over 3, 6, 9, and 12 months postoperatively by 58.7, 74.0, 82.9, and 89.4, respectively. Isokinetic peak torque deficit of the knee extensor muscles in 3, 6, 9, and 12 months postoperatively was 59.9%, 49.7%, 35.7%, and 28.1%, respectively. The rim plate-augmented separate vertical wiring with supplementary fixation for the treatment of patellar fracture associated comminuted inferior pole is effective and can be safely applied AO/OTA 34-C2 or C3 with favorable outcomes.

## Introduction

The surgical goal of repairing patella fracture is to restore the extensor mechanism while simultaneously achieving articular congruency. Several treatment options for patella inferior pole fracture have been reported in the literature. Various surgical techniques include separate vertical wiring^1^, SVW in combinations with cerclage wire^2^ or augmented Krackow suturing^3^, plate osteosynthesis^4^, suture button fixation^5^, partial patellectomy followed by patella tendon reconstruction using trans-osseous suture or anchors, among others^6–10^. However, there is a lack of study about surgical treatment for AO/OTA 43-C2, C3 associated comminuted inferior pole.

Despite the variety of treatment methods, comminuted inferior pole fractures of the patella remain difficult and technically demanding to achieve stable internal fixation because the characteristics of patella inferior pole fractures are extra-articular type combined with small fragments and comminution up to 83.3%^11–13^. The separate vertical wiring technique has been widely used and proven with satisfactory outcomes for these fractures^1,2^. Nevertheless, the surgical technique has limited capability to achieve stable fixation and can cause cutting through of wires with severe comminution in the sagittal and coronal plane of inferior pole patella fractures^8^. To support comminution fragments, Cho et al. modified the separate vertical wiring technique by adding a rim plate for comminuted inferior pole fracture of the patella^8^. Although the clinical result was favorable, the research has not evaluated functional outcomes thoroughly. To prove effectiveness of patient’s functional recovery and safety of surgical technique, it is necessary to assess the strength of the injured limb using the Cybex test^14^. There is insufficient data on the use of Cybex isokinetic testing to analyze the functional outcome after surgical treatment.

**Figure 1:**
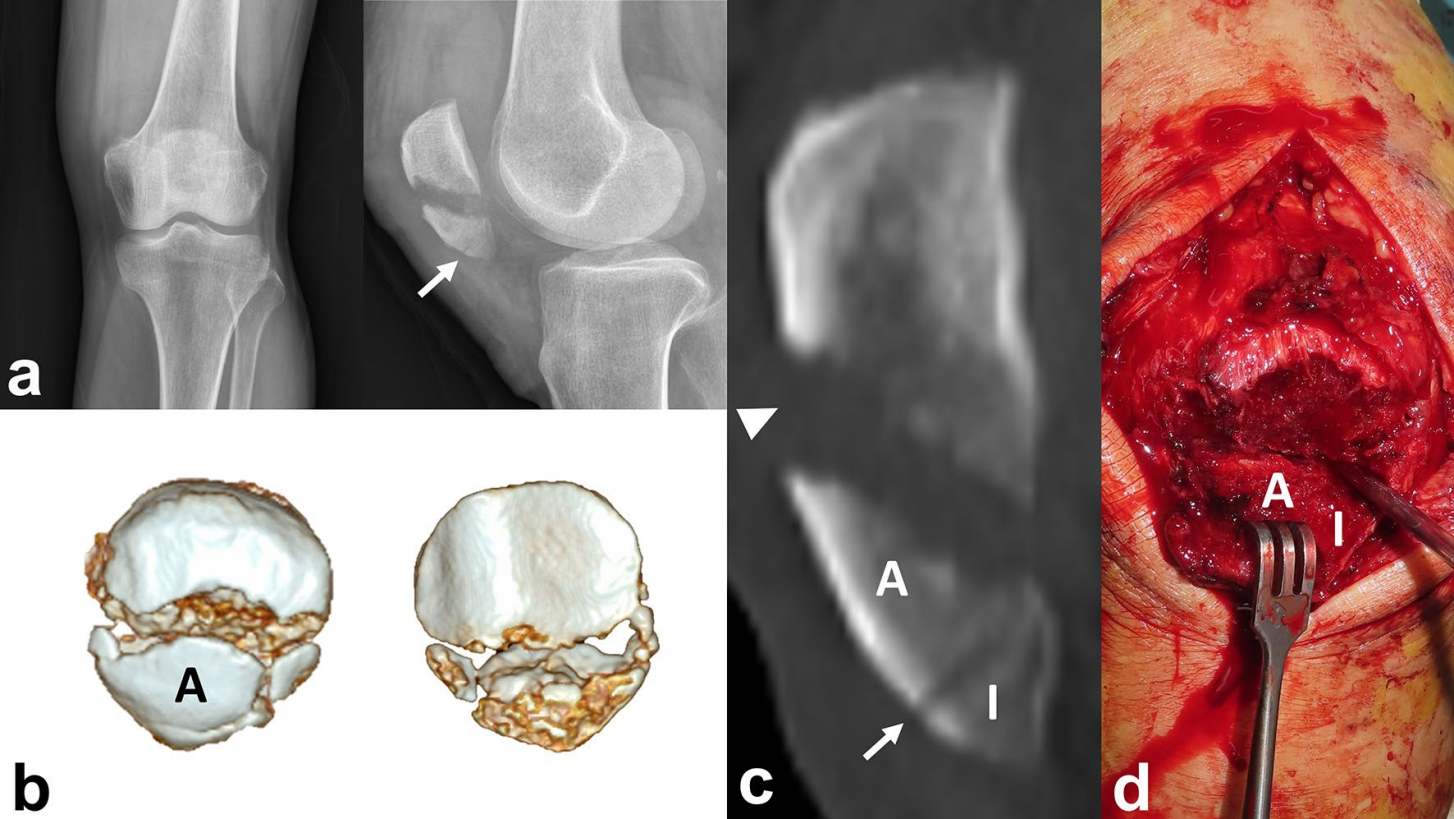
(**a**) A 40-year-old female patient diagnosed with a comminuted inferior pole fracture of patella which has anterior cortical breakage (white arrow). (**b**) An intact Anterior cortical split fragment with comminution. (**c**) The primary fracture line is located on middle level and the secondary fracture line is located on the lower articular boundary of the patella. (**d**) Intraoperatively, an Inferior pole fragment was separated from the anterior coronal split fragment.

**Figure 2:**
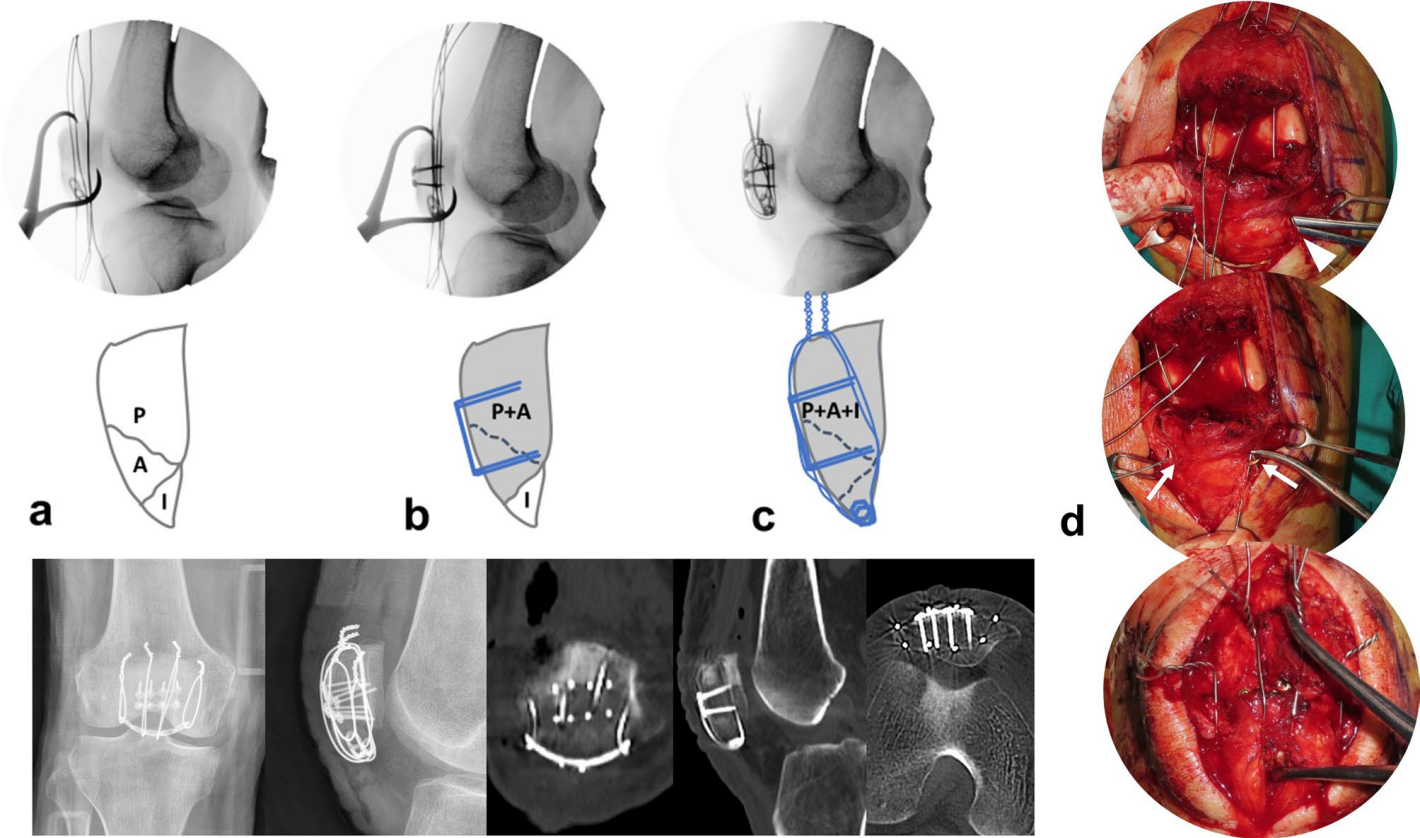
(**a**) A Proximal, Anterior, and Inferior pole fragment were reduced by reduction clamp with passed separate vertical wires engaged with a rim plate. (**b**) The Proximal and Anterior fragments were fixed by supplementary fixation using anterior cortical plate to be converted to type A from type C. (**c**) After conversion, rim plate augmented separated vertical wires were tightened. Postoperative radiographs show the rim-plate-augmented separate vertical wiring with supplementary fixation using anterior cortical plate. All identified fragments were anatomically stabilized. (**d**) The Mosquito was passed under the patellar tendon(white arrow head) before the rim plate passage. A medial and lateral wire(white arrow) went through both end holes of rim-plate. Middle wires were placed over the plate for wrapping.

Furthermore, a free articular coronal fragment or anterior coronal split fragment associated with inferior pole fracture is frequently found in three-dimensional morphological fracture mapping study^11^ (Fig. 1). These fractures should be managed with supplementary fixation. Besides AO/OTA 43-A1, there might be an expansion of the application of a novel surgical technique. When the fracture is classified with AO/OTA 43-C and has the secondary horizontal fracture line on the lower articular boundary of the patella, it can be converted to type A from type C by supplementary fixation, including plates or screws (Fig. 2a,b). After conversion, the treatment can be almost the same as AO/OTA 43-A1 (Fig. 2c,d). Thus, we have adopted rim-plate-augmented separate vertical wiring with supplementary fixation in the management of comminuted inferior pole fractures, AO/OTA 34-A1, C2 and C3 (Supplementary Fig. 1).

The purpose of this study is: (i) to present a novel technique of the rim-plate-augmented separate vertical wiring with supplementary fixation; (ii) to evaluate the radiologic and clinical outcomes, including functional score and Cybex test in treating patellar fractures associated comminuted inferior pole.

## Patients and methods

This study was a retrospective review of a prospectively collected cohort and was approved by the institutional review board (Dongsan Medical Center No. 2023-02-064). The inclusion criteria were as follows: (i) patients diagnosed with comminuted inferior pole fracture of the patella, which includes AO/OTA 34-A1; (ii) AO/OTA 34-C2 or C3, which has a primary or secondary horizontal fracture line on the lower articular boundary of the patella and can be converted to A1; (iii) patients treated operatively by using rim-plate-augmented separate vertical wiring with or without supplementary fixation; (iv) patients with a minimum of 1 year of follow-up; (v) patients with pre and post-operative radiographic evaluation including computed tomographic data; (vi) patients with measured functional score and Cybex test. The exclusion criteria were as follows: (i) pathologic fracture; (ii) open fracture with severe bone defect or extended soft tissue damage; (iii) pre-incident functional limitation on the knee joint. In current study, the surgical indication for AO/OTA 34-A1 which was surgically indicated due to the disruption of extensor mechanism and have comminution in inferior pole. Among the surgically indicated AO/OTA 34-C2 or C3, the fracture which have primary or secondary horizontal fracture line along the inferior border of articular facet and can be converted into extraarticular fracture by completing the fixation of coronal split and satellite fragment. The distinct fracture pattern from multi-fragmentary patellar fracture is described in detail on a patellar fracture mapping study^11^.

Between July 2013 and January 2020, 40 patients were diagnosed with comminuted inferior pole fracture of the patella with preoperative computed tomography and treated with rim-plate-augmented separate vertical wiring with supplementary fixation at two university medical centers. Among them, 3 patients did not meet the criterion of a minimum 1-year follow-up. A total of 37 patients (22 males and 15 females) were enrolled in the current study. Mean patients’ age was 51.4 ± 13.9 years (range 19–73). Mechanisms of injury included slip down in 30 patients, traffic accident in 4 patients, and fall down in 3 patients. All the patients were preoperatively evaluated with computed tomography (CT) scan to identify comminution of the inferior pole and the existence of free articular fragment. According to the inclusion criteria, patients with a primary horizontal fracture line on the articular surface were not enrolled. The average number of inferior pole fragments, which was defined as displaced more than 2 mm and separated by sagittal fracture line, was 3.1 ± 1.0 (range 1–5). The average amount of displacement between main fragments was 11.3 ± 6.0 mm (range 3–34) (Table 1).

**Table 1:** Patient demographics.

Variable	N (%)
Total number	37
Age (years)	
Mean ± SD (range)	51.4 ± 13.9 (19–73)
Sex	
Male	22 (59.5)
Female	15 (40.5)
AO/OTA classification	
A1	20 (54.1)
C2	11 (29.7)
C3	6 (16.2)
Mechanism	
Slip down	30 (81.1)
Fall down	3 (8.1)
Motor vehicle accident	4 (10.8)
Laterality	
Right	21 (56.8)
Left	16 (43.2)
Number of inferior pole fragments	
Mean ± SD (range)	3.1 ± 1.0
Displacement between main fragments (mm)	
Mean ± SD (range)	11.3 ± 6.0 (3–34)

All operations were performed by two orthopedic trauma surgeons (J. Cho and B. Kim) from university hospitals. Intraoperative data, including total operative time, was collected from medical records and fluoroscopic imaging in a picture archiving and communication system (PACS).

### Surgical technique

#### Surgical approach

**Figure 3:**
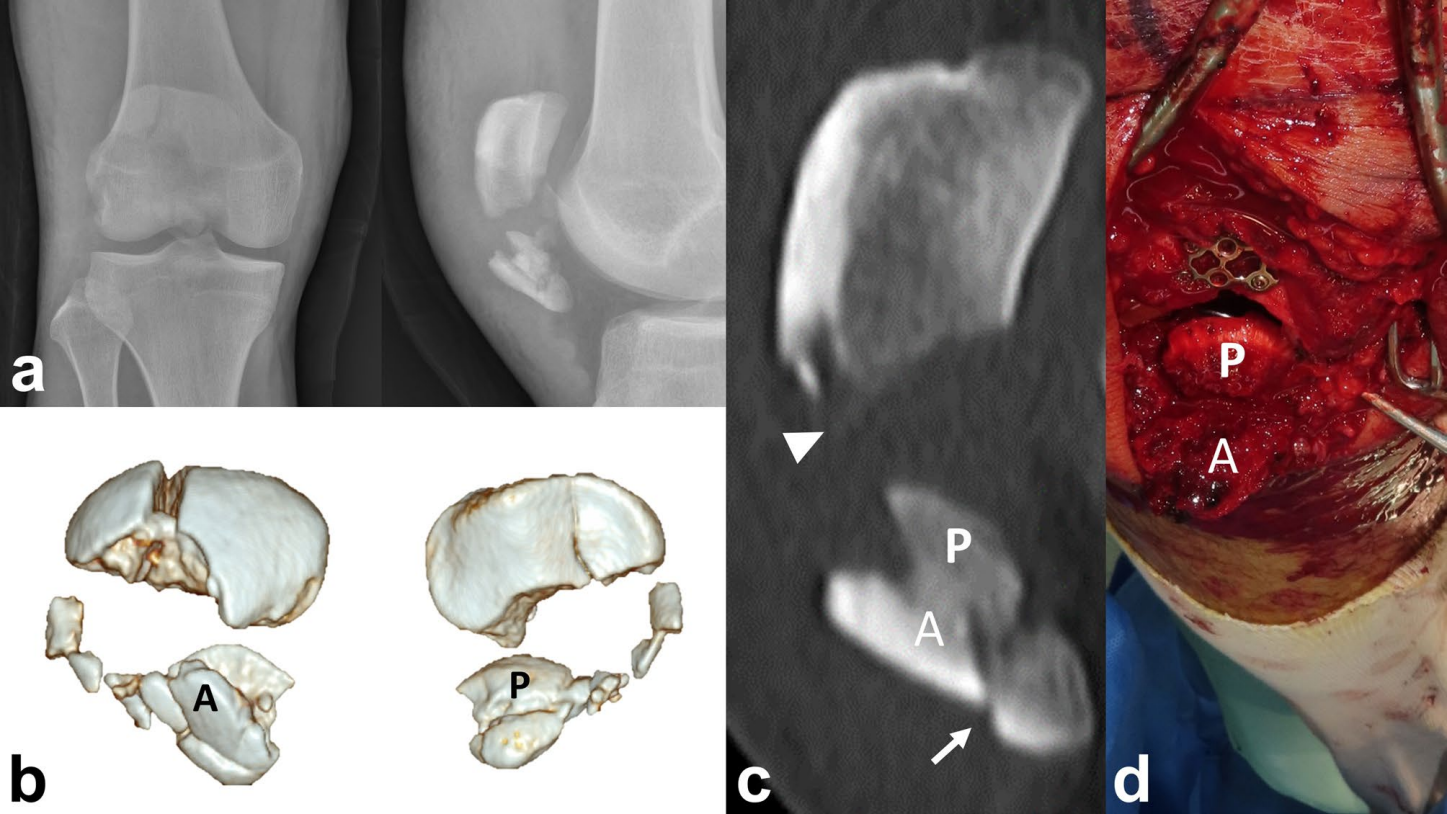
(**a**) A 20-year old male patient diagnosed with AO/OTA 34-C3 associated with a comminuted inferior pole fracture. (**b**) An Anterior cortical split and Posterior articular split fragment with inferior pole comminution. Also, a vertical split fracture on the proximal fragment was identified. (**c**) The primary horizontal fracture line is located on the middle level of the patella (arrow head), and the secondary fracture line is located on the lower articular boundary of the patella (white arrow). (**d**) Intraoperatively, those split fragments were separated.

Under spinal or general anesthesia, the patient was placed in the supine position with 20° flexion of the knee. In general, pneumatic tourniquet was applied on the proximal thigh and the injured leg was draped in a manner. After the inflation of a pneumatic tourniquet, a vertical midline incision, approximately 10 cm in length, was made on the anterior aspect of the knee from the lower half of the quadriceps tendon to the upper half of the patellar tendon. The anterior cortex and articular surface were examined to inspect concealed fracture comminution and free articular fragments (Fig. 3).

#### Sequence of procedure

**Figure 4:**
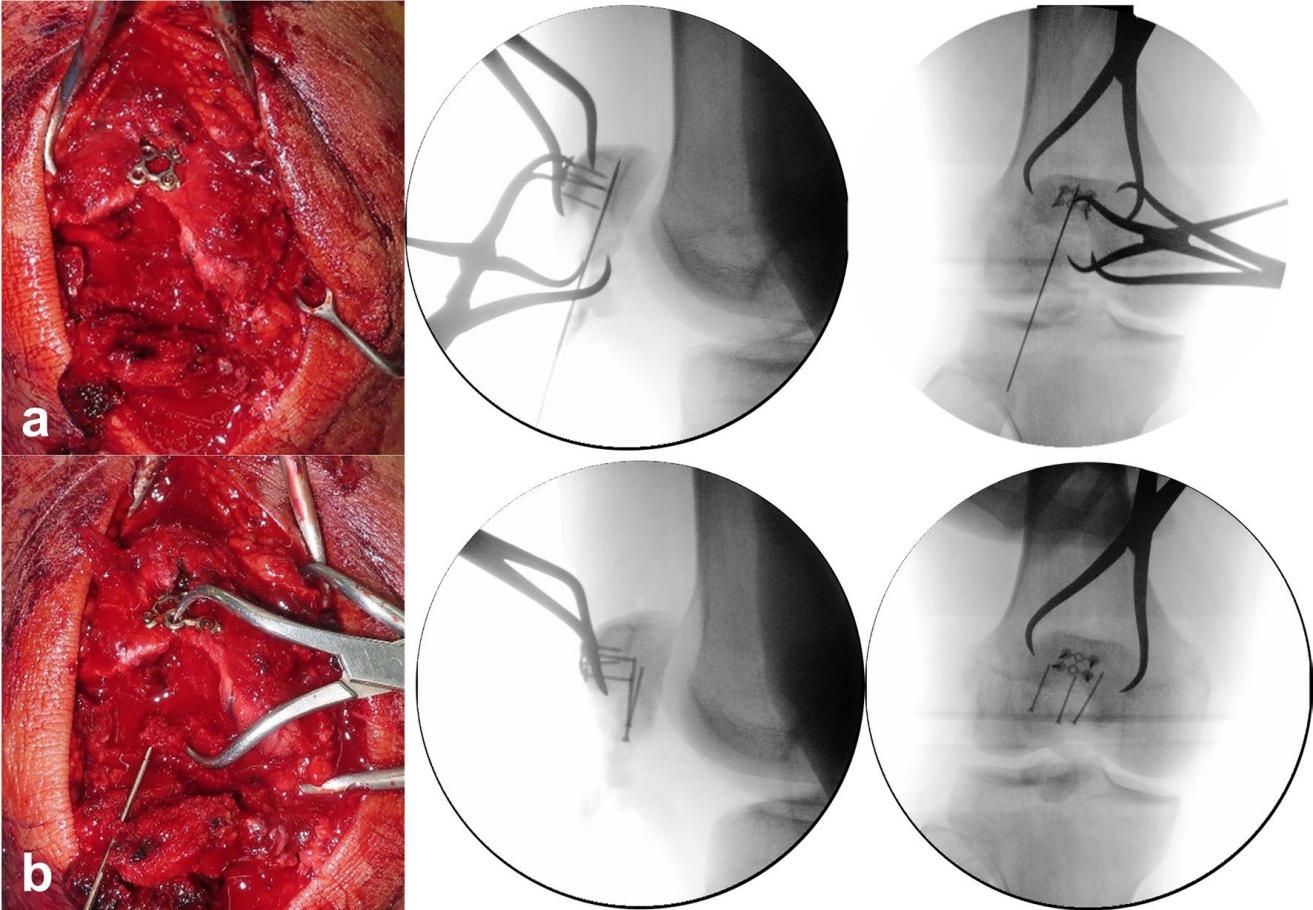
Supplementary fixation using (**a**) anterior cortical plate for a vertical split fracture on proximal fragment and (**b**) embedded screws for a posterior articular split fragment.

**Figure 5:**
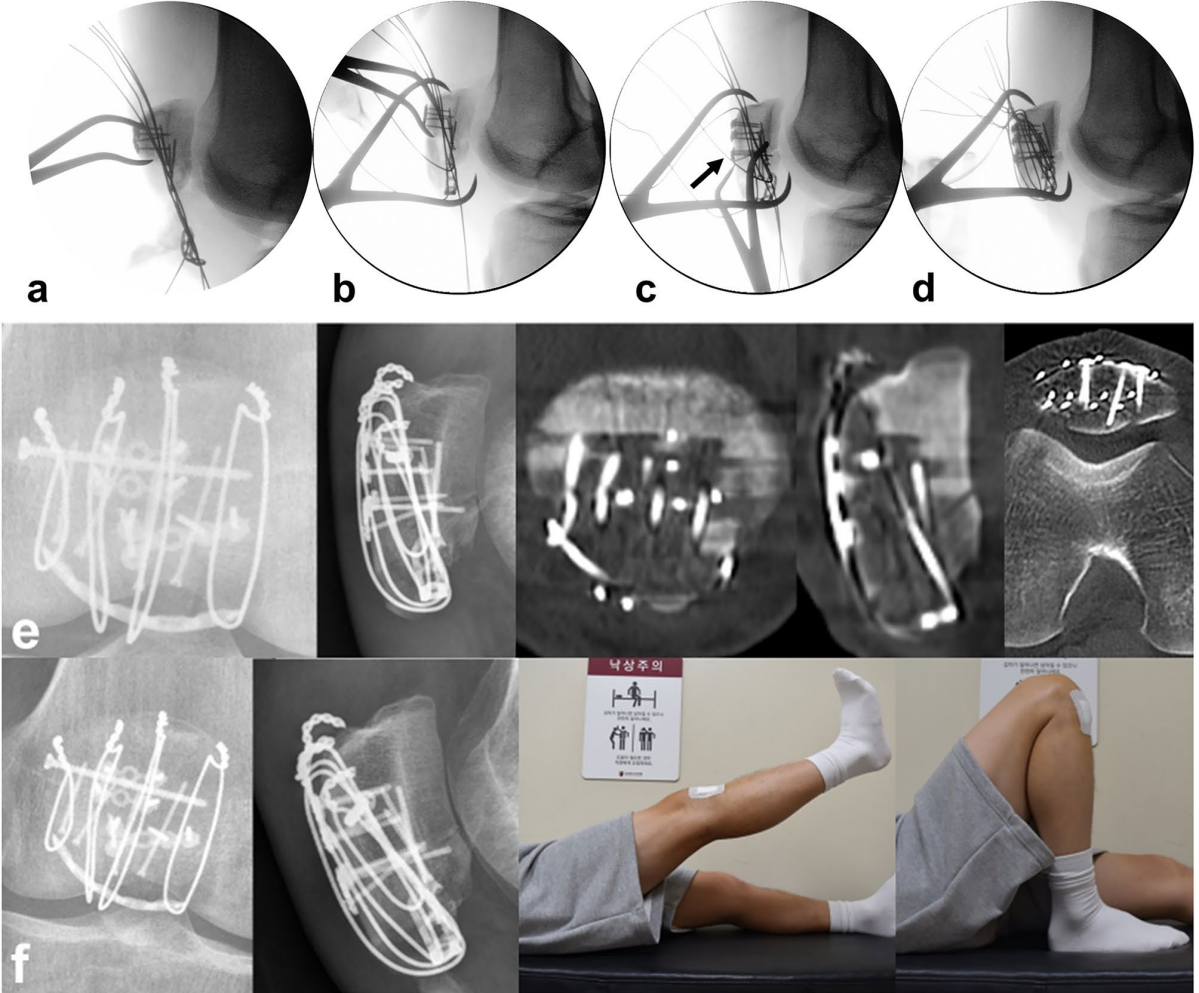
(**a**) Placement of rim plate and engagement of wires. (**b**) Reduction of proximal and distal fragments using reduction clamp. (**c**) An additional anterior cortical plate fixation for anterior cortical split fragment. (**d**) Tightening of vertical wires. (**e**) Postoperative radiographs show the rim-plate-augmented separate vertical wiring with supplementary fixation using two anterior cortical plates and embedded screws. All identified fragments were anatomically stabilized. (**f**) After 6 months, radiologic bony union was achieved and range of motion was fully recovered without extension lag.

i. *Conversion type of fracture “C” to “A” by supplementary fixation* The AO/OTA 34-C2 or C3 can be managed by screw or plate fixation in order to convert into AO/OTA 34-A1. After reduction with point reduction clamps, these fractures were fixed with 1.5 mm size plate (LCP; LCP Compact Hand, DePuy Synthes, USA) or embedded 1.5 mm screws for free articular fragments. (Fig. 4).
ii. *Placement of three or four 1.0 mm vertical separate wires at proximal fragment* Kirschner wire(1.4 mm) was passed between articular surface and anterior cortex of proximal fragment through the fracture gap in retrograde direction. After making a preliminary hole, 1.0 mm roll wires (~ 15 cm in length) were prepared and put into the holes. The proximal tip of the wires was taken out from quadriceps muscles through the stab incisions.
iii. *Passing the wires beneath distal fragments* A stab incision was made on the center of the patellar tendon. Distal ends of wires were passed beneath the distal fragments and taken out through the window on the patellar tendon.
iv. *Placement of rim plate and engagement of wires* Length and contour of a 1.5 mm or 2.0 mm plate (LCP; LCP Compact Hand, DePuy Synthes, USA) was tailored according to the width of the inferior pole fracture fragment. The rim plate was passed under the patellar tendon. A medial and lateral wire went through both end holes of rim-plate. Middle wire was placed over the plate for wrapping.
v. *Reduction of proximal and distal fragments* A pointed reduction clamp was utilized to reduce proximal and distal fragment. The inferior prong of the clamp was placed on the rim plate to prevent a split in the fracture comminution.
vi. *Tightening of vertical wires* While maintaining the reduction, the medial and lateral vertical wires were pulled anteriorly and tightened evenly at the anterosuperior aspect of the proximal fragment. After then, the middle wire was tightened to wrap the rim plate. The twisted tip of the wires was cut and buried under the quadriceps tendon to prevent irritation. (Fig. 5)

### Postoperative protocols

After surgery, patients were wrapped with cotton roll and elastic bandage. A hinged knee brace was employed, and a plan to start muscle strengthening exercises and continuous passive motion for 2–3 days post-operation was enacted. Patients were encouraged to start partial weight bearing (about 50% of their weight) 2 weeks after surgery, increasing to full weight 3–4 weeks after surgery. The range of motion was gradually increased and exercised with the goal of 90° flexion by 4 weeks post-operation, 120° flexion by 6 weeks post-operation, and full range afterward.

### Outcome measurement

The follow-up intervals from postoperative protocol are at 2 weeks and 1, 2, 3, 6, 9, 12 months. Routine plain radiographs were taken in anteroposterior (AP), lateral (LAT), and both obliques (OBL). The radiologic bony union was defined as the presence of bony trabecular continuity and the loss of fracture line. If the fracture line was ambiguously visible, computed tomography was utilized to confirm the union.

In order to evaluate functional outcomes, Cybex tests were performed, and Lysholm scores with a range of motion were checked at 3, 6, 9, 12 months^14–16^. Isokinetic concentric knee extension peak torque was measured using a Cybex II dynamometer. Before testing, the system was calibrated using standard weights. Flexion power and extension power were evaluated with an angular velocity of 60°/s^16^. Patients were seated with the hips at 90° and the back straight and were stabilized with body and thigh straps. The pad of the cruris was placed 3–4 cm superior to the medial malleolus. The uninjured limb was tested first. During the tests, all the patients were encouraged via visual and verbal stimuli. The proportional isokinetic knee extension peak torque deficit in the injured leg was calculated in relation to the uninjured limb. The results were classified as *good* when deficits were lower than 30%, *moderate* when between 30 and 44% and *poor* when exceeding 45%, based on the categorization of Levack et al.^17^.

### Ethical approval

All experimental protocols from this study were approved by Dongsan Medical Center, School of Medicine, Keimyung University, Institutional Review Board. All procedures and methods performed in studies involving human participants were in accordance with the ethical standards of the institution or practice at which the studies were conducted. (IRB number: Dongsan Medical Center No. 2023-02-064) Informed consent was obtained from all individual participants included in the study.

## Results

The rim-plate-augmented separate vertical wiring without supplementary fixation was applied on 20 patients (54%, 20/37) who were classified AO/OTA 34-A1. As a supplementary fixation for conversion from type C to type A, plate, embedded screw, or combination were used on 17 (46%, 17/37), 5(29%), 4(24%), and 8(47%) patients who were classified as AO/OTA 34-C2 or C3, respectively. The mean operation time was 93.5 ± 28.6 min (range, 34–195). A 6-hole 2.0mm plate was mostly used, and the average number of separate vertical wires was 3 (range 3–5).

In all patients, bony union was achieved at an average of 3.1 ± 1.4 months (range 2–6) after surgery. The mean follow-up duration was 32.8 ± 12.4 months (range 13–62). There was no patient with loss of reduction, metal failure, and infection during follow-up. There were 6 patients with implant irritation, 3 patients with patellar tendinitis and 4 patients who underwent implant removal surgery. The average final range of motion was 131.6 ± 7.2 degrees (range 120–140), and the average time to achieve the final range of motion was 3.7 ± 1.2 months (range 3–6) (Table 2).

**Table 2:** Surgical details and results.

Variable	AO/OTA 34-A (n = 20)	AO/OTA 34-C2, C3 (n = 17)	Total (n = 37)
Size of rim plate (number of holes)			
4	2 (10)	1 (5.9)	3 (8.1)
5	5 (25)	6 (35.3)	11 (29.7)
6	12 (60)	6 (35.3)	18 (48.6)
7	1 (5)	4 (23.5)	5 (13.5)
Supplementary fixation			
Plate		5 (29%)	
Screw		4 (24%)	
Combination		8 (47%)	
Operation time (min)			
Mean ± SD (range)	86.9 ± 25.3 (34–140)	101.4 ± 30.9 (68–195)	93.5 ± 28.6 (34–195)
Follow-up duration (month)			
Mean ± SD (range)	34.1 ± 10.5 (13–49)	31.3 ± 14.0 (15–62)	32.8 ± 12.4 (13–62)
Bony union	20 (100)	17 (100)	37 (100)
Union time (month)			
Mean ± SD (range)	2.7 ± 0.9 (2–6)	3.7 ± 1.7 (2–6)	3.1 ± 1.4 (2–6)
Complications			
Implant irritation	4 (20)	2 (12)	6 (16)
Metal failure	None	None	None
Patellar tendinitis	2 (10)	1 (6)	3 (8)
Implant removal surgery	2 (10)	2 (12)	4 (11)
Infection	None	None	None

The average of Lysholm knee scores gradually increased over 3, 6, 9, and 12 months postoperatively, 56.6 ± 14.9 (range 10–90), 72.5 ± 13.4 (range 20–95), 82.8 ± 8.1 (range 60–96) and 88.5 ± 7.6 (70–98) respectively. At the final follow-up, 17 patients (46%) had an excellent function, and 10 patients (27%) had good function.

The average isokinetic peak torque of the knee extensor muscles was significantly lower in the injured leg than in the uninjured leg, 3, 6, 9, and 12 months postoperatively. The isokinetic peak torque deficits were 60.1 ± 11.5 (%, range 30–79), 49.1 ± 16.9 (%, range 3–80), 35.5 ± 13.3 (%, range 2–60) and 26.9 ± 13.8 (%, range 3–58) respectively. According to criteria from Levack et al.^17^, 22 patients (59%) and 12 patients (32%) had good and moderate quadriceps strength, respectively (Table 3).

**Table 3:** Clinical outcome with Lysholm score and Isokinetic concentric knee extension peak torque deficit at angular velocity of 60°/s (Cybex test).

Variables	AO/OTA 34-A (n = 20)	AO/OTA 34-C2, C3 (n = 17)	Total (n = 37)
Final ROM			
Mean ± SD (range)	131.5 ± 6.5 (120–140)	131.8 ± 8.1 (120–140)	131.6 ± 7.2 (120–140)
Time to achieve final ROM (month)			
Mean ± SD(range)	3.8 ± 1.3 (3–6)	3.6 ± 1.2 (3–6)	3.7 ± 1.2 (3–6)
Lysholm score			
3 months			
Mean ± SD (range)	56.9 ± 9.7 (42–75)	56.4 ± 19.7 (10–90)	56.6 ± 14.9 (10–90)
6 months			
Mean ± SD (range)	72.8 ± 9.5 (48–88)	72.2 ± 17.2 (20–95)	72.5 ± 13.4 (20–95)
9 months			
Mean ± SD (range)	82.8 ± 6.3 (65–96)	82.8 ± 10.0 (60–95)	82.8 ± 8.1 (60–96)
12 months			
Mean ± SD (range)	88.0 ± 6.8 (75–97)	89.2 ± 8.6 (70–98)	88.5 ± 7.6 (70–98)
Cybex test			
3 months			
Mean ± SD (range)	59.3 ± 11.8 (30 ~ 78)	60.8 ± 11.6 (30 ~ 79)	60.1 ± 11.5 (30 ~ 79)
6 months			
Mean ± SD (range)	43.8 ± 16.3 (− 3 ~ 70)	53.5 ± 16.5 (9 ~ 80)	49.1 ± 16.9 (− 3 ~ 80)
9 months			
Mean ± SD (range)	32.7 ± 13.6 (2 ~ 60)	37.9 ± 12.8 (15 ~ 60)	35.5 ± 13.3 (2 ~ 60)
12 months			
Mean ± SD (range)	23.1 ± 13.8 (3 ~ 58)	30.2 ± 13.3 (8 ~ 54)	26.9 ± 13.8 (3 ~ 58)

A repeated measures ANOVA was performed to compare the clinical outcomes on each follow-up period. From the result, there was a statistically significant difference in the mean Lysholm score and Cybex test at least two different follow-up period that represents improvement in the clinical outcomes during the postoperative period (Table 4, Supplementary Fig. 2).

**Table 4:** Repeated measures ANOVA of Lysholm score and Cybex test.

Source	df	SS	MS	F	P
Lysholm score					
Group (G)	1	0.002	0.002	< 0.01	0.998
Test time (T)	3	21,728.02	7242.67	134.12	< 0.001*
G × T	3	19.04	6.35	0.12	0.950
Cybex test					
Group (G)	1	1253.49	1253.49	2.32	0.137
Test time (T)	3	23,791.62	7930.54	106.68	< 0.001*
G × T	3	328.77	109.59	1.47	0.226

Greenhous-Geisser Correction, Group: AO/OTA 34-A, AO/OTA 34-C2, C3; Test Time: Postoperative 3, 6, 9, 12 months; G × T: group × time; *p < 0.05.

## Discussion

The treatment of inferior pole patella fracture is challenging due to the inherent weakness of the bone and the comminution of the fragments, which interrupt firm fixation through ordinary fixation methods. There is a lack of consensus about the ideal fixation method for the inferior pole of patellar fractures.

Various methods have been demonstrated in the literature for the osteosynthesis of these fractures. The tension band wiring and screw fixations are not recommended because they cannot provide firm stabilization when the fragment is too small or comminuted^7,9,10^. Egol et al. reported 30.6% of patients required reoperation in the tension band wiring cohort. In their suture cohort, one patient had an initial failed fixation (7.6%)^18^. Specifically, reduction loss of distal pole fragments and wire cut-through can occur^3,8^. Thus, we concluded that a specific fixation method with modification and augmentation is mandatory to achieve successful osteosynthesis.

Yang and Byun et al. reported a separate vertical wiring for the fixation of comminuted fractures of the inferior pole of the patella with favorable clinical outcomes^1^. However, the ultimate load of this fixation system (250 N) is lower than the force loaded to the completely extended quadriceps muscle (316 N)^13^. Therefore, 4 weeks of long leg cast immobilization of the injured knee is required. Song et al. demonstrated augmentation with additional cerclage wire to separate vertical wiring to achieve a higher average ultimate failure load (325 N)^2^. However, the ultimate load before failure was still not high enough, and there might be concerns of fixative failure clinically due to eventual excessive contracture of the quadriceps. Cho et al. modified SVW technique by rim plate augmentation and reported successful treatment of comminuted inferior pole patella fracture^8^. The technique provides a metal template, which acts like a large washer, to prevent vertical wire cut-through where the comminutions exist on the sagittal plane. In addition, by engaging and tightening each vertical wire into the plate holes, the rim plate can evenly compress the fracture site. Even when the comminution is extremely severe, the rim-plate-augmented SVW can support the fragments by the rim plate which is caught into the tendinous junction of the inferior pole. So far, there is no biomechanical study of this construct that can prove its safety and effectiveness. However, we believe that the estimate of biomechanical strength is not inferior to simple SVW or SVW augmented with cerclage wiring. Especially in the severe comminution model, it can provide superior strength than those aforementioned techniques. However, there are some disadvantages compared with other surgical techniques. Firstly, the surgical procedure can be relatively longer than simple methods. Secondly, the cost might be increased due to 2.0 mm rim plate and supplement implants. Finally, it is more difficult to remove implants after inserting numerous screws and plate. The embedded screw cannot be noninvasively removed.

In our current study, we expanded the indication of the surgical technique, that is AO/OTA 34-C2 or C3 with the secondary horizontal fracture line on the lower articular boundary of the patella. By using supplementary fixation, including separate screws, embedded screws, and anterior small size plate, the above C-type fractures can be converted to A-type. From the three-dimensional morphological fracture mapping study, several convertible fracture figurations were identified, including a free articular coronal fragment or an anterior cortical split fragment associated with an inferior pole fragment^11^. Furthermore, vertical split fracture of the proximal fragment can also be converted to A-type after being fixed with 2.7 mm cortical screw or small size plate. Radiologic results of AO/OTA 34-C2, C3 showed no reduction loss with 100% union rate. This favorable outcome was not inferior to the results of AO/OTA 34-A1 from the current study. There were 4 patients with implant irritation associated with tip of wire loop and 2 patients associated with palpable rim plate around patellar tendon. Among them, 2 patients had the implants removed as the mild uncomfortableness from implant irritation. Four patients underwent the implants removal as a result of social and psychologic reasons in oriental cultures. However, there was no wire breakage and wire cut-through.

The current study is the first report on the use of Cybex isokinetic testing to evaluate the outcome of rim plate-augmented SVW technique for inferior pole fracture of the patella. There is a lack of evidence regarding the recovery strength of the extensor mechanism or quadriceps after internal fixation of the patella. The result of the Cybex test showed that the average isokinetic peak torque of the extensor muscles was significantly lower in the injured leg, especially 3 and 6 months post-operation. However, more than 90% of patients had *good* and *moderate* quadriceps strength 12 months post-operation. In the early period, the extensor muscle function is generally impaired due to pain, muscle injury, and associated knee joint structure damage^16^. However, the full range of knee joint motion was almost completed in the early period of therapy, so immediate active knee motion exercise is very important. This finding indicates that the secure stabilization of internal fixation is mandatory for early active rehabilitation. Several previous studies show that the SVW technique alone was not yet known or considered to be suitable for early active exercise. In the current study, all of the patients started muscle strengthening exercises and continuous passive motion 2–3 days post-operation to accomplish satisfactory clinical outcomes. After a minimum of 12 months of follow-up, there was no metal failure or reduction loss. From our point of view, the fixation construct is strong enough and safe for immediate rehabilitation. Also, we believe that the result of Cybex test after internal fixation for inferior pole fracture of the patella is worth utilizing as a reference for further research in the future.

The current study has several limitations. Firstly, the surgical technique was not verified by biomechanical assessment. Theoretically, the ultimate load before the failure of our construct is considered to be superior to the original SVW. To prove mechanical stability, biomechanical analysis is required in comminuted inferior pole fracture of patella model. Secondly, a relatively small number of AO/OTA 34-C2 and C3 was not enough to represent the safety and efficacy of the application of this surgical technique. Although the radiologic and clinical outcomes were favorable and comparable to AO/OTA 34-A1, the outcome was not statistically evaluated. The study design was a retrospective investigation without comparison, and other associated variables were not controlled. Furthermore, the severity of articular fracture on AO/OTA 34-C2 and C3 can affect the clinical outcome, including Lysholm score and Cybex test. Also, it can cause persistent pain, knee joint stiffness or post-traumatic osteoarthritis. Fourth, there might be a selection bias. The AO/OTA classification of 34-C2 and C3 contains diversiform fracture morphology, including associated inferior pole comminuted fracture. We enrolled specific convertible fracture figurations of C-type, which has the secondary horizontal fracture line on the lower articular boundary of the patella. By using supplementary fixation, we could convert the fracture type from C to A. However, in some cases, the fracture line was not clearly identified, and the articular boundary of the patella was too irregular to be determined. Lastly, unified supplementary fixation methods were not conducted for C to A converting procedure. Despite the above limitations, we believe that the rim-plate-augmented separate vertical wiring with a supplementary fixation for the treatment of patellar fracture associated comminuted inferior pole is effective and can be safely applied to certain fracture configurations of AO/OTA 34-C2 or C3 with favorable radiologic and clinical outcomes.

## Conclusion

The findings of the current study support that the rim-plate-augmented separate vertical wiring with a supplementary fixation for the treatment of patellar fracture associated comminuted inferior pole was effective and can be safely applied to AO/OTA 34-C2 or C3, which has the secondary horizontal fracture line on the lower articular boundary of the patella, resulting in favorable radiologic and clinical outcomes without any major complications.

### Supplementary Information

Supplementary Legends.

Supplementary Figure 1.

Supplementary Figure 2.

## Data Availability

The datasets generated during and/or analysed during the current study are available from the corresponding author on reasonable request.
