# Supplementary material for: Clinical outcome of rim-plate-augmented separate vertical wiring with supplementary fixation for the treatment of patellar fracture associated comminuted inferior pole
Source: Sci Rep. 2023 Aug 18;13:13430. doi: 10.1038/s41598-023-40417-w (PMC10439214; doi:10.1038/s41598-023-40417-w)
Supplement: Supplementary file 1 — Supplementary Legends. [file 41598_2023_40417_MOESM1_ESM.docx]

**Supplementary legends**

**Supplementary Figure 1**.

**(a)** Radiographs from last follow-up at post-operative 24 months and **(b)** CT scan images at post-operative 3 months of the 40-year-old female patient, who was diagnosed with a comminuted inferior pole fracture of patella which has anterior cortical breakage, showed radiologic bony union.

**Supplementary Figure 2.**

The mean Lysholm score and Cybex test from postoperative 3, 6, 9, 12 months.
