# Supplementary figures and images for: Clinical outcome of rim-plate-augmented separate vertical wiring with supplementary fixation for the treatment of patellar fracture associated comminuted inferior pole
Source: Sci Rep. 2023 Aug 18;13:13430. doi: 10.1038/s41598-023-40417-w (PMC10439214; doi:10.1038/s41598-023-40417-w)

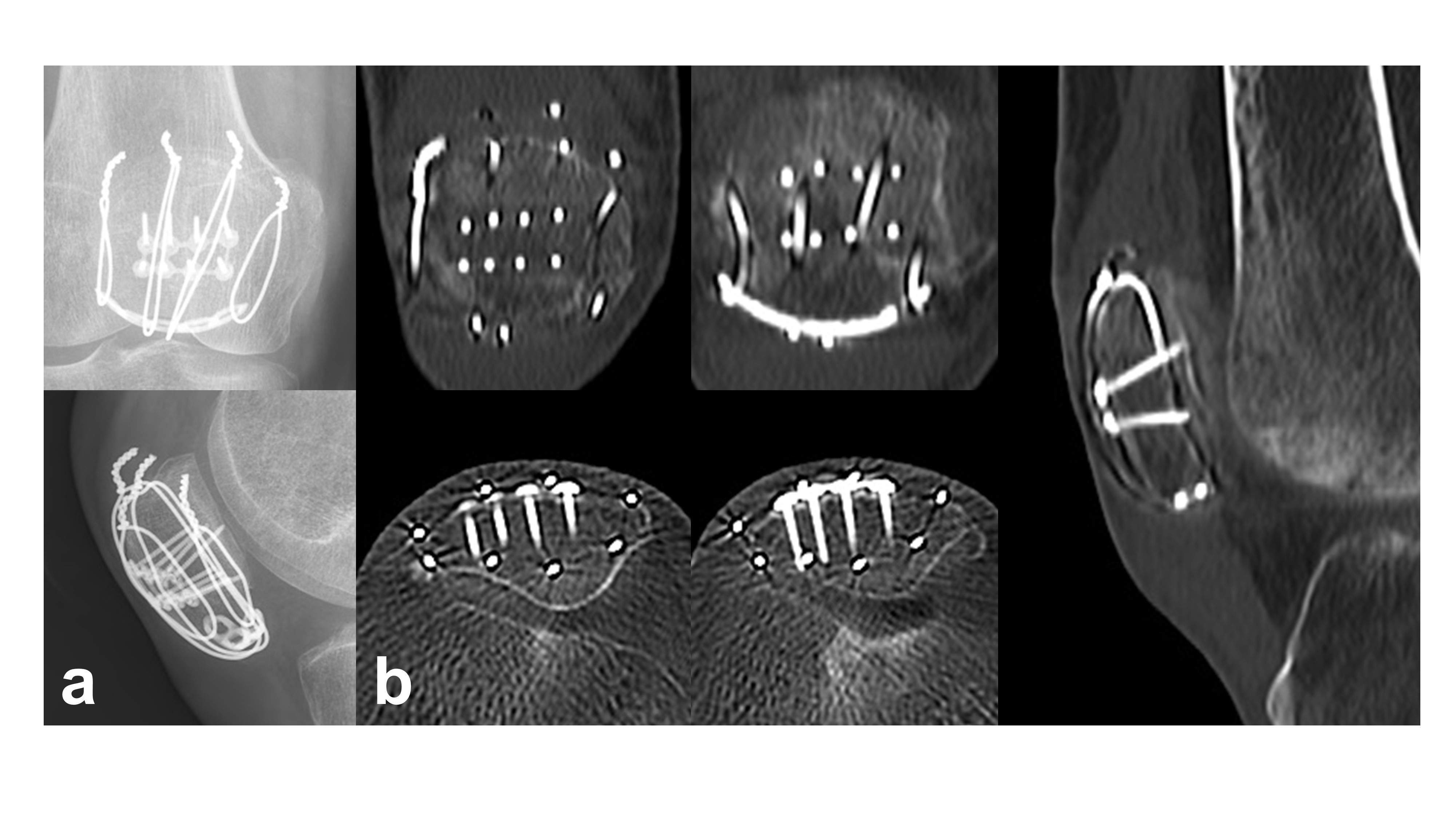

Supplement: Supplementary file 2 — Supplementary Figure 1. [file 41598_2023_40417_MOESM2_ESM.jpg]

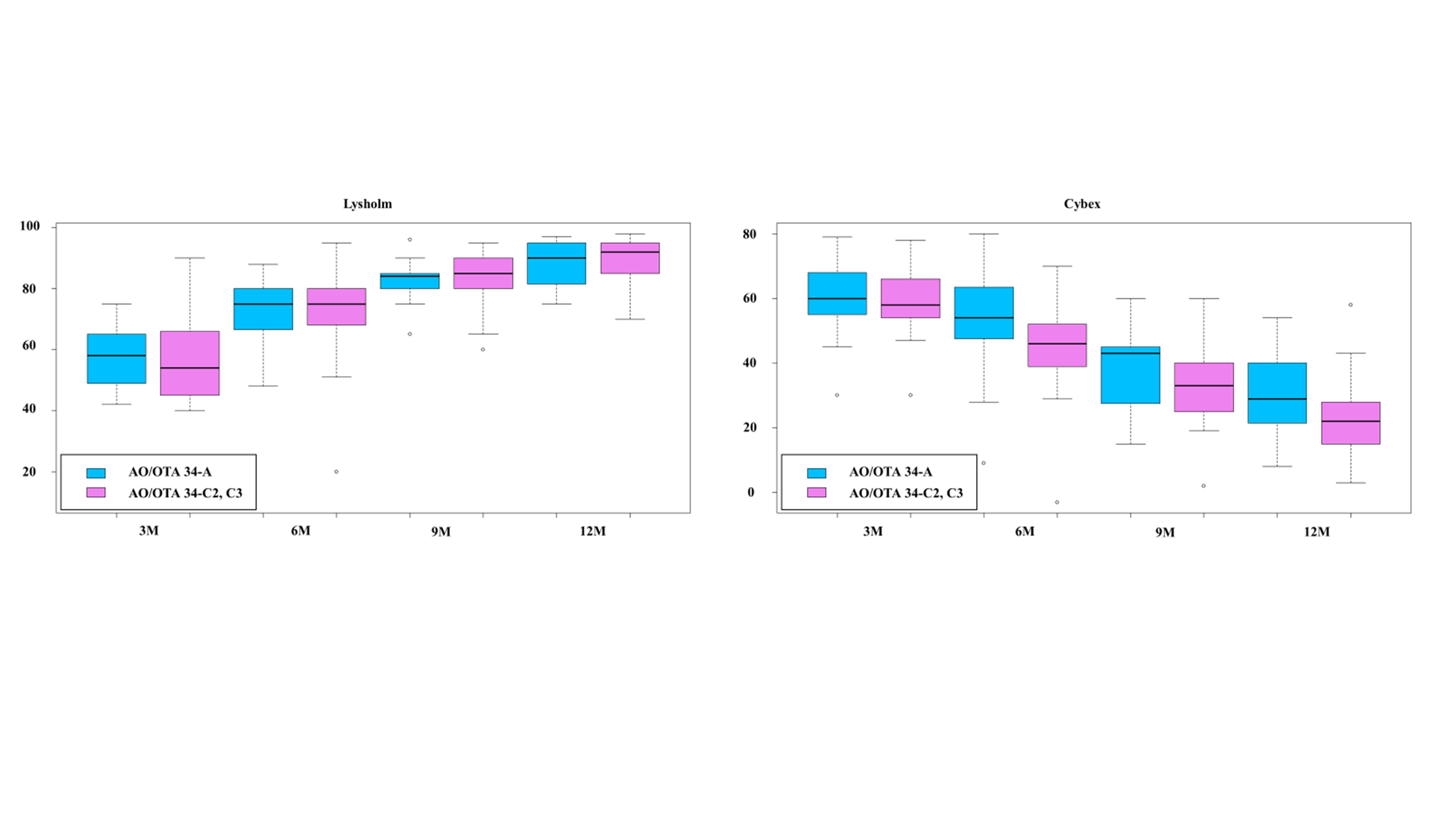

Supplement: Supplementary file 3 — Supplementary Figure 2. [file 41598_2023_40417_MOESM3_ESM.jpg]
